# Food and health promotion literacy among employees with a low and medium level of education in the Netherlands

**DOI:** 10.1186/s12889-021-11322-6

**Published:** 2021-06-30

**Authors:** Hanne C. S. Sponselee, Willemieke Kroeze, Maartje P. Poelman, Carry M. Renders, Kylie Ball, Ingrid H. M. Steenhuis

**Affiliations:** 1 Department of Health Sciences, Faculty of Sciences, VU University Amsterdam and Amsterdam Public Health Research Institute, 1081 HV Amsterdam, Netherlands; 2 Care for Nutrition and Health Group, School of Nursing, Christian University of Applied Sciences, 6717 JS Ede, The Netherlands; 3 Chair Group Consumption and Healthy Lifestyles, Wageningen University and Research, 6700 EW Wageningen, The Netherlands; 4 Institute for Physical Activity and Nutrition, Deakin University, Geelong, VIC Australia

**Keywords:** Food literacy, Self-perceived food literacy, Health literacy, Health promotion literacy, Level of education, Workplace, Prevention, Socioeconomic health inequities

## Abstract

**Background:**

Prior research indicates a positive association between socioeconomic position and health literacy levels. We hypothesize comparable socioeconomic gradients for food literacy. This study aims to determine the level of self-perceived food literacy and health promotion literacy among adults with a low and medium level of education and from various subgroups, as well as the association between these food and health literacy levels. Furthermore, this study aims to explore the associations of self-perceived food literacy (SPFL) and health promotion literacy (HPL) in BMI.

**Methods:**

A cross-sectional study was conducted among employees with a low and medium level of education. Descriptive analyses were performed to compute SPFL and HPL levels. Analyses of variance were performed to test differences between subgroups. The correlation between SPFL and HPL was computed by Pearson’s r. Multivariate linear regression analyses were used to explore 1) the association between SPFL and HPL adjusted for demographic characteristics 2) the associations between SPFL and HPL in BMI.

**Results:**

The majority (63.1%) of all participants (*n* = 222) scored low on SPFL and 34.5% scored inadequate or problematic on HPL. No significant educational or weight-status differences were found in SPFL or HPL levels. On most levels, women compared to men and older compared to younger employees scored significantly higher. A small positive correlation between the two mean levels was found, *r* = .25, *P* < .001 (*n* = 203). Multivariate linear regression analyses showed a significant association between SPFL and HPL (*B* = .31, 95% CI = .15–.48). No significant associations between SPFL and HPL in BMI were found.

**Conclusions:**

This study suggests there is room for improvement in SPFL and HPL among adults with a low and medium level of education. Future research should consider comparing low and middle socioeconomic with high socioeconomic groups when exploring food and health literacy. Regarding health promotion activities for adults with a low and medium level of education, it is recommended to focus on improving both food and health literacy. Furthermore, more research is needed to explore direct proxies of weight-status to better understand the role of food and health literacy in overweight patterns.

## Background

The burden of non-communicable diseases (NCDs) and associated risk factors are major health challenges of the twenty-first century [1]. Among the most important modifiable risk factors for developing NCDs are overweight and obesity [1]. Behavioural change interventions which focus on promoting healthy dietary and physical activity behaviours can be effective in achieving weight loss [2]. However, these interventions are often most effective among people with a high socioeconomic position (SEP) [3] compared to those with a low SEP, whereas obesity and unhealthy lifestyle behaviours are mostly observed among people with a low SEP [4, 5]. These behaviours are found to substantially contribute to socioeconomic health inequities [6].

The relationship between lifestyle behaviours such as eating behaviour, on the one hand, and health, overweight, obesity and the prevalence of NCDs on the other is well established [7–9]. Eating behaviour is complex and can be influenced by a wide range of factors [10]. One important factor is the food environment, which currently promotes the consumption of energy-dense, nutrient-poor foods [11]. The extent to which people are capable of making healthy food choices in this environment is covered by the concept of food literacy. Food literacy can be described as the interconnected knowledge, skills and behaviours which are essential for planning, managing, selecting, preparing and eating foods in order to meet needs and determine food intake [12]. Additional to this individual perspective, recent attempts have been made to more comprehensively define food literacy by including the role of food systems on access and adherence to healthy eating [13]. Furthermore, a wide range of research describes the conceptual association between food literacy and health, well-being and nutrition behaviour [12, 14–19]. To the contrary, the empirical association between food literacy and diet is limited [20]. Similarly, the association between food literacy, individual and collective well-being has not been researched extensively yet [15, 20]. However, the limited existing evidence suggests that higher levels of food literacy may be associated with healthier food consumption [21] and that improving food literacy behaviours may lead to higher diet quality [22].

Food literacy is a specific form of the broader concept of health literacy [17], defined by Sørensen et al. (2012) as ‘people’s knowledge, motivation and competences to access, understand, appraise and apply health information in order to make judgments and take decisions in everyday life concerning healthcare, disease prevention and health promotion to maintain and improve quality of life throughout the life course’ [23]. Low health literacy levels have been found to be associated with negative health outcomes such as increased overall mortality [24], less use of preventive services [25], and increased body mass index (BMI), overweight and obesity [26]. In Europe, 47.6% of the population show inadequate or problematic levels of health literacy [27]. In the Netherlands, these inadequate or problematic levels are found in more than one in three adults (36.4%) [28].

Health literacy levels have regularly been reported to be unequally distributed throughout society. Regarding gender, the evidence for the association with health literacy is inconsistent. Some studies show that the level of health literacy in women is to a certain extent higher than in men [29–33] although others show no differences [34, 35]. Evidence for the role of age in predicting health literacy seems to be consistent, with older age associated with low health literacy levels [36–38]. However, there might be differences in the direction of this role between health literacy dimensions [31]. Regarding socioeconomic differences, people with a low SEP often show lower levels of health literacy compared to people with a high SEP [39]. Understanding and measuring health literacy levels is important when considering the prevention of chronic diseases [40] because these levels are strongly related to health and well-being [24, 26]. Specifically, the domains of health promotion and disease prevention within the concept of health literacy [23] are expected to provide valuable insights into health promotion activities in a prevention context. In this study, these domains will be collectively referred to as ‘health promotion literacy’.

As food literacy is closely linked to health literacy [17] and people with a low SEP tend to consume lower-quality diets compared to people with a high SEP [5], we hypothesize that people with a low level of education will also have lower levels of food literacy in line with observed socioeconomic gradients in health literacy [26, 39]. Furthermore, there is a lack of knowledge regarding the empirical association between food literacy and health literacy as stated before. Although the concepts of food literacy and health literacy are closely linked [17], theoretical differences exist. Health literacy has a relatively greater focus on health information [23], while food literacy is described relatively more commonly in terms of skills and behaviour [12]. Therefore, determining whether and to what extent the association between food and health literacy exists empirically will add to our understanding of the way in which health promotion activities should be directed.

This study aims to determine the level of self-perceived food literacy and health promotion literacy among adults with a low and medium level of education and from various subgroups (i.e. based on gender, age, level of education and BMI), as well as the association between these levels. Furthermore, it explores the associations between self-perceived food literacy and health promotion literacy in BMI.

## Methods

### Design, study population and recruitment

The workplace was chosen as the setting for this cross-sectional study because it is part of a research project which aims to influence the behaviour and environments of people with a low and medium level of education. Inclusion criteria for the study population were being 18 or older, being employed, living in the Netherlands, understanding the Dutch language at a sufficient level and having a low or medium level of education (i.e. with secondary vocational education as the highest level of education).

Regarding recruitment, job sectors which mainly employ people with a low and medium level of education [41] were selected first. Second, companies throughout these job sectors were selected by means of purposive sampling. A list was made of companies across the Netherlands which were expected to employ at least 30 persons with a low or medium level of education. Third, 124 companies throughout 9 of the 12 provinces of the Netherlands were emailed an invitation to participate in this study. If they did not answer, the researcher (HS) called them within a week to explain the study in more detail and to invite them to participate. Twenty companies responded that they were not willing to participate for several reasons (e.g. corporate reorganization processes, involvement in other studies, workload or no interest in preventive health). Eighty-nine companies did not elaborate on their reasons for non-participation or could not be reached. The contact persons (e.g. employers or human resource managers) of fifteen companies which were interested in participating were contacted by telephone. Eight companies in a variety of job sectors – civil engineering (*n* = 1), healthcare (*n* = 3), construction (*n* = 1), public sector (*n* = 1), infrastructure (*n* = 1) and logistics (*n* = 1) – were ultimately willing to participate. The contact persons of these eight companies approached their employees based on the inclusion criteria and by using flyers provided by the researcher (HS).

### Data collection and procedure

Data were collected using a questionnaire that was filled in by participants at their workplace. First, the questionnaire was pilot-tested among a small convenience sample of employees with a low level of education (*n* = 4) in a real-life work setting. The researcher asked the questions and the employee answered according to the ‘thinking aloud’ method [42]. Their insights suggested that the items of the health literacy scales should be shortened and their comprehensibility improved. Therefore, a Dutch linguistic company, also involved in the development of the Self-Perceived Food Literacy Scale (SPFLS) [21], converted the health promotion literacy items to ‘B1’ language level, a level that is generally understood by 95% of people [43]. This adaptation was made in consultation with the researcher to retain the items’ connotations.

Participants filled in the questionnaire either in an individual setting or in a group setting due to practical reasons (e.g. time constraints set by the employer and the employees’ type of job). In the individual setting, participants completed the questionnaire in a private room using a computer assisted modality while the researcher (HS) was present. Participants who had difficulty reading were able to indicate their preference for the researcher to read the questions aloud. In the group setting, participants completed the questionnaire using a paper and pencil modality while the researcher (HS) and other participants were present in the same room. All participants provided written informed consent. Questionnaire completion took 15 to 30 min. Participants were compensated with a healthy snack.

### Measurements

#### Self-perceived food literacy scale

The Self-Perceived Food Literacy Scale (SPFLS) is a validated, theory-driven, expert-based instrument which measures self-perceived food literacy (SPFL) with respect to healthy eating among adults, using B1 language level [21]. The overall scale shows good reliability (Cronbach’s *α* = 0.83) and is comparable to the reliability found in our sample (Cronbach’s *α* = 0.84). Furthermore, convergent, divergent and criterion validity have been indicated [21]. The SPFLS consisted of 29 items with five-point Likert answering scales (from 1 = ‘No, never’ to 5 = ‘Yes, always’). A higher mean SPFL score indicated a higher level of SPFL [21]. The items covered the following eight subscales: Food Preparation Skills (e.g. *‘Are you able to alter a recipe yourself?’*), Resilience and Resistance (e.g. ‘*Are you able to eat healthily when you feel stressed?’*), Healthy Snack Styles (e.g. ‘*Do you eat fruit as a snack?’*), Social and Conscious Eating (e.g. *‘Do you find it important to eat dinner at the same time if you are with others?*’), Examining Food Labels (e.g. *‘Do you check the nutritional labels of products for calories, fat, sugar or salt content?’*), Daily Food Planning (e.g. *‘If you have something to eat, do you take account of what you will eat later that day?’*), Healthy Budgeting (e.g. *‘Do you purchase healthy foods, even if they are a bit more expensive?’*) and Healthy Food Stockpiling (e.g. *‘Do you have 4 or more bottles of sugar-sweetened beverages or lemonade containing sugar in stock?’*). These subscales corresponded to the variables used in the analyses of this study. Items which required reversed scoring were recoded. No cut-off point for missing values was described by the SPFLS developers [21] and therefore only cases without missing values were used to calculate mean SPFL.

In order to interpret mean SPFL levels, mean SPFL was divided into the two categories low (1.00–3.49) and high (3.50–5.00). The connotation of the response option 3.00 = ‘Sometimes I do, sometimes I do not’ arguably does not represent having a high level of SPFL while the option 4.00 = ‘Yes, most of the time’ does. Therefore, the cutoff point of 3.49 in the middle of those response options was chosen. This procedure was also conducted in a German study, to our knowledge the first and only study that applied the SPFLS to a general population including people with a low level of education [44].

#### Health promotion literacy

Since our study was conducted within the context of prevention and health promotion, we used the subscales ‘Health Promotion’ and ‘Disease prevention’ of the short European Health Literacy Survey Questionnaire (HLS-EU-Q16) [45, 46] in Dutch to measure ‘health promotion literacy’ (HPL). We used the items of the HLS-EU-Q16 subscales instead of the original HLS-EU-Q47 [45] for pragmatic reasons (e.g. to make sure that the completion time of the total questionnaire would be feasible). The HPL scale (HPLS) showed good reliability (Cronbach’s *α* = 0.83). Convergent validity was indicated by a positive correlation (*r* = .25, *P* < .001, *n* = 203) between the HPLS and SPFLS. Furthermore, face validity was indicated by a thorough review process of the maintenance of the items’ connotations after conversion to B1 language level, by the first author and three of the co-authors.

The subscale of Health Promotion measures the ability to access, understand and process information on determinants of health in the social and physical environment. This subscale was composed of four items (e.g. *‘If family or friends give you advice about health, do you understand that advice?’*), which were answered on a four-point Likert scale (from 1 = ‘No, very poorly’ to 4 = ‘Yes, very well’). The subscale of Disease Prevention assesses the ability to access, understand, process and apply information on risk factors for health. This subscale was composed of five items (e.g. *‘Do you understand information about unhealthy behaviour? For example about smoking, physical activity or drinking alcohol?’),* which were answered on a four-point Likert scale (from 1 = ‘No, I understand it very poorly’ to 4 = ‘Yes, I understand it very well’). The answer option ‘I don’t know’ was not used as it is only used when administered orally by an interviewer [45].

Mean HPL was calculated based on the nine items of both subscales. Furthermore, HPL categories were calculated. The principles of the sum scoring procedure of the HLS-EU-Q16 [46] from which the HPL items were derived, were applied to calculate HPL categories. First, each item was dichotomized by scoring the answer options 1 and 2 (‘Very difficult’ and ‘Difficult’) as 0 and the answer options 3 and 4 (‘Easy’ and ‘Very easy’) as 1. Subsequently, this resulted in a sum score ranging from 0 to 9. In line with the HLS-EU-Q scoring procedures in which a health literacy score can only be computed for respondents who answered at least 80% of the items [27, 46], only respondents who completed at least 8 out of 9 items were considered. Cut-off points were based on the distribution of sum scores in the HLS-EU-Q16 categories. The sum score ranges from 0 to 16 in the HLS-EU-Q16, of which 50% is attributed to the category inadequate (0–8), 25% to the category problematic [9–12] and 25% to the category sufficient [13–16, 46]. This distribution was applied to the HPL sum score ranging between 0 and 9, which resulted in the three HPL categories inadequate (0–5), problematic [6, 7] and sufficient [8, 9].

#### Characteristics of participants regarding demographics and BMI

Demographic information was obtained from each participant concerning gender, age and highest level of education. Furthermore, self-reported height (centimetres when not wearing shoes) and self-reported weight (kilograms) were obtained. BMI was calculated by dividing the weight in kilograms by the square of the height in meters. Furthermore, it was divided in three weight-status categories: Healthy weight (18.50–24.99), overweight (25–29.99) and obese (≥ 30.0).

#### Level of education

Level of education was divided into two levels according to the Dutch standard classification of education [47]: low (those whose highest level of education was that of secondary vocational education level 1) and medium (those whose highest level of education was that of secondary vocational education level 4).

### Data analyses

Data were analysed in SPSS Statistics 26.0. The presence of outliers among the main variables was tested. One outlier on BMI was found and subsequently converted to missing data. Furthermore, one BMI value was below the healthy weight range (i.e. < 18.5 kg/m^2^ [48]). This value was converted to missing data as *n* = 1 was not sufficient to make it a separate category. Missing values (i.e. 19 for SPFL and 2 for HPL) were left as ‘missing’ without excluding cases.

The sample was described by performing descriptive analyses. The mean values for SPFL, HPL, and their subscales as well as the distribution across categories were calculated using descriptive analyses. Analyses of variance (ANOVA) were performed to test the difference in mean SPFL and HPL between the subgroups gender, age, level of education and BMI. First, assumptions for normality were assessed and met. A Tukey’s post hoc test was used to pairwise compare the three BMI categories. Accordingly, significant between-group differences were explored. The correlations between SPFL, HPL and the subscales were computed by the Pearson’s correlation test. A significance level of 0.05 was used (2-sided). Pearson’s correlation coefficients were interpreted as small (≥ .10 *r* < .30), moderate (≥ .30 *r* < .50) or large (*r* ≥ .50) [49]. The association between SPFL and HPL was explored by multivariate linear regression analyses, with SPFL as outcome variable and demographic characteristics as covariates (i.e. gender, age and level of education). The assumptions were tested and met. No statistically significant interaction between demographic characteristics and HPL was found, hence analyses were not stratified. The relationships between SPFL and HPL in BMI respectively were also explored using multivariate linear regression analyses. First, assumptions were tested and met. BMI was the outcome variable. Subsequently, adjustments for demographic characteristics (i.e. gender, age and level of education) as covariates were applied. No statistically significant interaction between demographic characteristics and either HPL nor SPFL were found, hence analyses were not stratified.

## Results

In total, 222 participants met our inclusion criteria. As shown in Table 1, the majority of the sample were men (61.1%) and aged 40 or older (60.2%).

**Table 1:** Characteristics of participants including demographics, BMI, SPFL levels and HPL levels

Characteristic	*n*		Subgroup	*n (*%)
Gender	221			
			Men	135 (61.1%)
			Women	86 (38.9%)
Age (*M* ± *SD*)	216	42.6 ± 13.0		
			< 40 years	86 (39.8%)
			≥ 40 years	130 (60.2%)
Level of education	222			
			Low	109 (49.1%)
			Medium	113 (50.9%)
BMI (*M* ± *SD*)	211	25.9 ± 4.3		
Weight-status			Healthy weight	94 (44.5%)
			Overweight	80 (37.9%)
			Obese	37 (17.5%)
SPFL (*M* ± *SD*)	203	3.37 ± .47		
Range		1.00–5.00		
			Low	128 (63.1%)
			High	75 (36.9%)
HPL (*M* ± *SD*)	220	3.11 ± .40		
Range		1.00–4.00		
			Inadequate	21 (9.5%)
			Problematic	55 (25%)
			Sufficient	144 (65.4%)

### Descriptive and univariate analyses

Mean SPFL and the distribution over low and high levels can be found in Table 1. SPFL was highest for the Healthy Budgeting subscale (3.96 ± .77) and lowest for the Examining Food Labels subscale (2.27 ± 1.19) (Table 2). The scores on the overall SPFL scale and on seven out of the eight SPFL subscales were significantly higher amongst women than men. To illustrate, on the subscale Food Preparation Skills the mean for women was 3.91 (*SD* = .69) and 3.57 for men (*SD* = .83) (*F* (1,211) = 3.07, *P* = .002). Solely the scores on the Examining Food Labels subscale did not differ between men and women (*F* (1,216) = 3.07, *P* = .081). The overall level of SPFL was significantly higher among participants aged 40 or older than among participants aged under 40 (*F* (1,195) = 11.63, *P* = .001). Significantly higher mean scores were found among participants aged 40 or older on the Daily Food Planning (*F* (1,213) = 5.67, *P* = .018), Social and Conscious Eating (*F* (1,211) = 20.00, *P* = <.001), and Resilience and Resistance (*F* (1,205) = 10.33, *P* = .002) subscales. No significant differences were found between participants with a low or medium level of education regarding mean SPFL (*F* (1,201) = .021, *P* = .885) or subscales. Similarly, no significant differences were found between the weight-status categories regarding mean SPFL (*F* (2,191) = .73, *P* = .484) or subscales.

**Table 2:** Comparison between subgroups with respect to SPFL and HPL levels

	Total													
		Gender (*n* = 221)			Age (*n* = 216)			Education (*n* = 222)			BMI (*n* = 211)			
		Men	Women		< 40 years	≥ 40 years		Low	Medium		Healthy	Overweight	Obese	
1. SPFL														
*M*	3.37	3.26	3.57		3.25	3.47		3.37	3.38		3.36	3.34	3.46	
*SD*	.47	.42	.49		.50	.43		.46	.48		.48	.45	.51	
*P-value*				<.001			.001			.885				.484
1a. Examining Food Labels														
*M*	2.27	2.16	2.45		2.19	2.34		2.20	2.33		2.17	2.33	2.55	
*SD*	1.19	1.11	1.30		1.18	1.20		1.15	1.23		1.19	1.15	1.25	
*P-value*				.081			.364			.423				.239
1b. Food Preparation Skills														
*M*	3.70	3.57	3.91		3.61	3.80		3.60	3.78		3.82	3.57	3.65	
*SD*	.80	.83	.69		.86	.71		.73	.84		.77	.81	.81	
*P-value*				.002			.082			.094				.112
1c. Healthy Food Stockpiling														
*M*	2.97	2.85	3.21		2.80	3.11		2.94	3.00		2.90	2.98	3.19	
*SD*	1.11	1.06	1.13		1.14	1.07		1.03	1.18		1.18	1.02	1.15	
*P-value*				.018			.051			.688				.417
1d. Daily Food Planning														
*M*	3.01	2.89	3.23		2.80	3.16		3.06	2.96		2.89	3.15	3.09	
*SD*	1.10	1.06	1.12		1.19	1.02		1.05	1.14		1.14	1.00	1.10	
*P-value*				.024			.018			.519				.272
1e. Healthy Budgeting														
*M*	3.96	3.85	4.14		3.85	4.04		3.93	3.98		3.94	3.92	4.12	
*SD*	.77	.83	.61		.82	.73		.81	.73		.72	.76	.90	
*P-value*				.005			.077			.677				.396
1 f. Social and Conscious Eating														
*M*	3.85	3.72	4.04		3.54	4.03		3.88	3.82		3.70	3.93	4.02	
*SD*	.82	.85	.73		.87	.72		.83	.81		.91	.69	.85	
*P-value*				.005			<.001			.589				.077
1 g. Resilience and Resistance														
*M*	3.60	3.52	3.72		3.43	3.70		3.64	3.56		3.60	3.59	3.58	
*SD*	.61	.58	.65		.65	.57		.62	.60		.68	.57	.55	
*P-value*				.018			.002			.370				.985
1 h. Healthy Snack Styles														
*M*	3.14	3.04	3.33		3.16	3.16		3.11	3.18		3.14	3.06	3.24	
*SD*	.83	.85	.77		.81	.86		.83	.84		.85	.79	.87	
*P-value*				.013			.977			.545				.512
2. HPL														
*M*	3.11	3.08	3.15		3.02	3.17		3.12	3.10		3.14	3.06	3.19	
*SD*	.40	.41	.38		.42	.38		.38	.42		.37	.48	.33	
*P-value*				.182			.010			.693				.237
2a. Health Promotion														
*M*	3.10	3.10	3.10		3.05	3.14		3.08	3.13		3.16	3.07	3.08	
*SD*	.44	.44	.44		.50	.40		.42	.46		.42	.50	.41	
*P-value*				.893			.183			.456				.427
2b. Disease Prevention														
*M*	3.12	3.07	3.20		3.01	3.19		3.16	3.08		3.12	3.05	3.29	
*SD*	.43	.45	.39		.43	.42		.42	.44		.38	.51	.35	
*P-value*				.021			.002			.180				.022

*P-value* is significant at the 0.05 level (2-tailed)

Mean HPL and the distribution over inadequate, problematic and sufficient can be found in Table 1. Women scored significantly higher than men on the Disease Prevention subscale (*F* (1,213) = 5.39, *P* = .021) (Table 2). Participants aged 40 or older scored significantly higher on HPL (*F* (1,212) = 6.85, *P* = .010) and the Disease Prevention subscale (*F* (1,208) = 9.52, *P* = .002) than participants under the age of 40. No significant difference was found between participants with a low level of education and participants with a medium level of education regarding mean HPL (*F* (1,218) = .157, *P* = .693) or subscales. Regarding HPL and weight-status, a significant difference was found for the Disease Prevention subscale (*F* (2,204) = 3.87, *P* = .022). A Tukey’s post hoc test showed that participants with obesity scored significantly higher on the Disease Prevention subscale than participants in the overweight subgroup (*P* = .016). There was no significant difference between participants with a healthy weight and participants with overweight (*P* = .568) or between participants with a healthy weight and obesity (*P* = .103).

### The association between SPFL and HPL

Pearson’s r indicated a small positive correlation between SPFL and HPL, *r* = .25, *P* < .001 (*n* = 203). A small positive correlation was also found between SPFL and the Health Promotion subscale of HPL, *r* = .24, *P* = .001 (*n* = 201). Likewise, a small positive correlation was found between SPFL and the Disease Prevention subscale of HPL, *r* = .23*, P* = .001 (*n* = 201). Furthermore, multivariate linear regression analyses showed a significant association between SPFL and HPL (*B* = .31, 95% CI = .15–.48), after adjusting for the covariates gender, age and level of education (Table 3). This indicated that on average an increase of one point in HPL corresponded to an increase in SPFL of .31 points.

**Table 3:** Linear regression model for SPFL as dependent variable and HPL as independent variable

	Unstandardized Coefficients		Standardized Coefficients			95% confidence interval for B	
	B	Std. Error	Beta	t	Sig.	Lower Bound	Upper Bound
HPL	.31	.08	.25	3.73	<.001	.15	.48

Note: SPFL and HPL are presented after adjusting for the covariates gender, age and level of education

### Multivariate analyses for BMI

Multivariate linear regression analyses showed no significant associations between SPFL and HPL in BMI, after adjusting for the covariates gender, age and level of education (Table 4).

**Table 4:** Linear regression model for BMI as dependent variable, SPFL and HPL as independent variables

	Unstandardized Coefficients		Standardized Coefficients			95% confidence interval for B	
	B	Std. Error	Beta	t	Sig.	Lower Bound	Upper Bound
SPFL	.45	.66	.05	.69	.494	−.85	1.76
HPL	.17	.73	.02	.23	.815	−1.28	1.62

Note: SPFL and HPL are presented after adjusting for the covariates gender, age and level of education

## Discussion

The aim of this study was to determine the levels of self-perceived food literacy (SPFL) and health promotion literacy (HPL) among adults with a low and medium level of education and from various demographic subgroups, as well as the association between these levels. Furthermore, it aimed to explore the relationship between SPFL and HPL in BMI. The results indicate that most adults in our sample had a low level of SPFL and that a third had inadequate or problematic HPL levels.

We observed that women scored higher on the majority of the SPFL subscales compared to men. In terms of age, participants aged 40 or older scored significantly higher on SPFL and HPL than participants aged under 40. We found no educational or weight-status differences in the mean values of the overall SPFL scale and its subscales. Likewise, no educational difference was found for HPL. Regarding the Disease Prevention subscale of HPL, participants with obesity scored significantly higher than participants with overweight. The association between SPFL and HPL was positive, though small and also present after adjusting for gender, age and level of education. Finally, no relationships were found between SPFL and HPL in BMI.

We observed that women on average had higher SPFL compared to men, except for the subscale ‘Examining Food Labels’. Comparable differences were observed in prior research suggesting that women have a healthier diet than men [50] and a healthier diet appears to be related to higher levels of food literacy [21]. Furthermore, women have been found to spend more time cooking than men [51, 52]. Additionally, women appear to be more involved in weight control and to have stronger beliefs about healthy food consumption compared to men [53]. Therefore, women may be more engaged in acquiring knowledge and skills related to healthy eating. These findings may explain the gender differences observed in the SPFL levels in our sample.

Regarding age, we found positive associations with higher SPFL and HPL while other studies seem to consistently find opposite results for health literacy [36–38]. However, earlier research suggests that the direction of this association may vary between health literacy dimensions [31]. Therefore, our findings for HPL may be explained by the fact that it solely covers two domains of health literacy (i.e. health promotion and disease prevention) which seem to increase with age in our sample.

We found no differences in the mean values of SPFL, HPL and their subscales between adults with a low and medium level of education, though we expected to find an educational difference in SPFL and HPL based on previous studies in food literacy and health literacy [29, 34, 39]. However, these previous studies often compared low with high levels of education and operationalized the factor of level of education differently. We would suggest that the absence of an educational difference in our sample indicates that our sample does not differ in SPFL and HPL levels based on having a low or medium level of education.

The correlation between SPFL and HPL was positive, though small. Earlier research also indicated a positive association between food literacy and health literacy [15]. Moreover, the association between SPFL and HPL was still significant after adjusting for gender, age and level of education though it remained rather small. Our findings indicate that improving SPFL or HPL does not necessarily imply that the other concept will improve to the same extent. Therefore, health promotion activities such as lifestyle interventions which focus on stimulating healthy dietary behaviour among adults with a low and medium level of education should consider paying attention to both concepts. It furthermore suggests that SPFL and HPL are positively related in an empirical way, in addition to the theoretical association. Additionally, the results suggests that the relationship between SPFL and HPL exists independently of the demographic characteristics observed in our sample.

Furthermore, the absence of associations between SPFL and HPL in BMI was unexpected. The absence of weight-status differences in our sample may be explained by the fact that BMI can be influenced by a broad variety of factors. Our SPFL and HPL measures may not have been comprehensive and direct enough to indicate an association. Low health literacy has in fact been linked to an increased BMI in earlier research [26, 54]. SPFL has been previously linked to healthier food consumption [21] but not directly to BMI, factors such as vegetable intake [55] and fast food consumption [56] have been linked to BMI. Therefore, the inclusion of more direct proxies of SPFL such as food intake might have indicate BMI differences. More comprehensive and direct factors should therefore be explored when aiming to understand the role of food and health literacy in BMI.

One limitation of this study was that the purposive sampling method might have induced selection bias as companies’ contact persons who agreed to participate, might have already been engaged in health promotion activities for their employees. In addition, employees completed the questionnaire at their workplace in a setting in which the researcher was present. This might have induced social desirability. However, this presence also assured comprehensibility about the aim and content of the questionnaire. Another limitation was that the original SPFLS was validated in a sample with an above-average rather than a below-average level of education compared to the general Dutch population. Regarding the Health Promotion and Disease Prevention subscales of HPL, no validation in Dutch has been performed yet. However, both the SPFLS [21] as well as the HPL subscales were used after conversion to B1 language level and are therefore expected to be suitable for people with a low and medium level of education. Finally, a recent study concluded that combining self-reporting and objective measures for understanding health literacy could contribute to understanding health literacy more broadly [57]. Therefore, the self-reporting nature of SPFL and HPL should be noted. SPFL and HPL levels describe the participants’ ideas about their eating and health behaviours rather than their objective behaviours. Although these results are valuable in the broader understanding of food and health literacy, the generalizability of the findings is unknown and should therefore be interpreted cautiously.

One strength of this study was that we derived two subscales to measure HPL from the HLS-EU-Q16, which is expected to be suitable for determining health literacy in populations with limited literacy [58]. Therefore, we expect that the two measured subscales derived from the HLS-EU-Q16 were appropriate for our sample. Another strength was that we simplified these items to B1 language level and that certain items were provided with explanatory text, as is recommended for use within groups who are expected to show limited literacy levels [58]. Finally, the majority of the sample were men (61.6%), which is a strength as men are often underrepresented in research regarding food literacy aspects such as food purchase and cooking [59].

### Recommendations

Our results suggest that there is room for improvement in both SPFL and HPL levels among adults with a low and medium level of education, because the majority of our sample had a low SPFL level and a third had inadequate or problematic HPL levels. A contribution to this improvement can be made by addressing causal determinants of health literacy. General literacy is one of the best indicators of health literacy [60, 61] and therefore adapting health information to the general literacy levels (i.e. reading and writing) of this group may be a step towards this improvement.

Future research should consider exploring differences in food and health literacy between adults with a low and medium level of education on the one hand and adults with a high level of education on the other hand. Comparing low and medium educated groups with high educated groups might add to our understanding of socioeconomic trends in food and health literacy. Subsequently, it might contribute to our knowledge of ways to reduce socioeconomic health inequities. Furthermore, it is recommended to consider food and health literacy in health promotion activities not only for groups with a low but also with a medium level of education.

Another direction of future research is to further explore the relationship between food literacy, health literacy and BMI. Based on this study, it is recommended to investigate food and health literacy in ways that more directly influence BMI. This might provide us with a better understanding of the role of food and health literacy in weight-status.

## Conclusions

In conclusion, the majority of our sample scored low on SPFL and a third scored inadequate or problematic on HPL. Regarding subgroups, women scored higher on the majority of the SPFL subscales (7 out of 8) compared to men. No differences in SPFL or HPL were found between groups with a low and medium level of education. In addition, SPFL and HPL were shown to be positively associated, to a small extent. HPL was associated with SPFL after adjusting for demographic characteristics. No relationships were found between SPFL and HPL in BMI. Regarding future research, the comparison of low and middle SEP groups with high SEP groups when exploring food and health literacy levels should be considered. Additionally, future research should explore more direct proxies of weight-status to better understand the role of health and food literacy in overweight patterns.

## Data Availability

The datasets used and/or analysed during the current study are available from the corresponding author on reasonable request.

## Abbreviations

ANOVA: Analyses of variance
BMI: Body mass index
HPL: Health promotion literacy
HLS-EU-Q: European Health Literacy Survey Questionnaire
HLS-EU-Q16: Short European Health Literacy Survey Questionnaire-16
HLS-EU-Q47: European Health Literacy Survey Questionnaire-47
NCDs: Non-communicable diseases
SEP: Socioeconomic position
SPFL: Self-perceived food literacy
SPFLS: Self-Perceived Food Literacy Scale
